# *Bordetella pseudohinzii* targets cilia and impairs tracheal cilia-driven transport in naturally acquired infection in mice

**DOI:** 10.1038/s41598-018-23830-4

**Published:** 2018-04-09

**Authors:** Alexander Perniss, Nadine Schmidt, Corinne Gurtner, Kristina Dietert, Oliver Schwengers, Markus Weigel, Julia Hempe, Christa Ewers, Uwe Pfeil, Ulrich Gärtner, Achim D. Gruber, Torsten Hain, Wolfgang Kummer

**Affiliations:** 1 Institute of Anatomy and Cell Biology, German Center for Lung Research (DZL), Excellence Cluster Cardio-Pulmonary System (ECCPS), Justus-Liebig-University Giessen, Giessen, Germany; 2 Institute of Hygiene and Infectious Diseases of Animals, Justus-Liebig-University Giessen, Giessen, Germany; 3 Institute of Veterinary Pathology, Freie Universität Berlin, Berlin, Germany; 4 Institute for Medical Microbiology, Justus-Liebig-University Giessen, Giessen, Germany; 5 Bioinformatics and System Biology, Justus-Liebig-University Giessen, Giessen, Germany; 6 German Center for Infection Research (DZIF), Partner Site Giessen-Marburg-Langen, Giessen, Germany; 7 Central Experimental Animal Facility, Justus-Liebig-University Giessen, Giessen, Germany

## Abstract

Several species of the Gram-negative genus *Bordetella* are the cause of respiratory infections in mammals and birds, including whooping cough (pertussis) in humans. Very recently, a novel atypical species, *Bordetella pseudohinzii*, was isolated from laboratory mice. These mice presented no obvious clinical symptoms but elevated numbers of neutrophils in bronchoalveolar lavage fluid and inflammatory signs in histopathology. We noted that this species can occur at high prevalence in a mouse facility despite regular pathogen testing according to the FELASA-recommendations. Affected C57BL/6 J mice had, in addition to the reported pulmonary alterations, tracheal inflammation with reduced numbers of ciliated cells, slower ciliary beat frequency, and largely (>50%) compromised cilia-driven particle transport speed on the mucosal surface, a primary innate defence mechanism. In an *in vitro*-model, *Bordetella pseudohinzii* attached to respiratory kinocilia, impaired ciliary function within 4 h and caused epithelial damage within 24 h. Regular testing for this ciliotropic *Bordetella* species and excluding it from colonies that provide mice for lung research shall be recommended. On the other hand, controlled colonization and infection with *Bordetella pseudohinzii* may serve as an experimental model to investigate mechanisms of mucociliary clearance and microbial strategies to escape from this primary innate defence response.

## Introduction

Several *Bordetella* species are the cause of respiratory infections in mammals and birds, including whooping cough (pertussis) in humans. *B. pertussis*, the typical pathogen of this disease, is commonly classified as a “classical” *Bordetella* species, together with two other relevant human respiratory pathogens, *B. parapertussis* and *B. bronchiseptica*^1^. “Non-classical” *Bordetella* species include the closely related species (a) *B. avium*, the causative agent of bordetellosis in birds^2,3^, (b) *B. hinzii*, a commensal organism in the respiratory tract of poultry and an opportunistic pathogen in humans^4–6^, and (c) the most recently identified *B. pseudohinzii*, so far found only in mice^7–10^. *B. pseudohinzii* has been isolated for the first time from laboratory-raised mice. It is very closely related to *B. hinzii*, with 3,206 genes present in the genomes of both species, 570 being specific to *B. hinzii*, and 390 genes contained in the genome of *B. pseudohinzii*, but not *B. hinzii*^8^. Until recently, routine diagnostic tests were unable to discriminate between the two. Thus, it has been discussed that previous reports on the prevalence of *B. hinzii* in animal facilities and associated histopathological changes in the lung^11,12^ might in fact have dealt with *B. pseudohinzii*^8,9^. In ten out of twelve reported cases of mice which were PCR-positive for *B. pseudohinzii* in faecal pellets, an increased percentage of neutrophils in the bronchoalveolar lavage (BAL) fluid was observed, although the mice had no clinical signs of respiratory infection. Still, it was suggested to consider this species as a confounding organism in murine models of pulmonary disease with the consequence of colony screening^9^. The current FELASA (Federation for Laboratory Animal Science Associations) recommendations for the health monitoring of mouse colonies in experimental units do not list *B. pseudohinzii* in their health report form^13^, but the AALAS (The American Association for Laboratory Animal Science)/FELASA working group refers to *B. hinzii* as an “exotic agent” that “should be mentioned when found”^14^.

In the course of experiments designed to study the mechanisms that regulate mucociliary clearance, a mechanism that eliminates inhaled particles from the airways, we encountered single murine tracheas with unusually low cilia-driven particle transport speed (PTS). This was observed both at baseline and after stimulation with acetylcholine and ATP, known activators of ciliary beat frequency (CBF)^15^. Although these mice were derived from a specified-pathogen free (SPF) facility and had no overt clinical signs of respiratory illness, we subjected samples including oropharyngeal swabs, tissue samples from trachea and lung from all mice used for PTS experiments to routine microbiological diagnostics. As a common denominator, non-classical *Bordetella*, not listed in the current FELASA recommendations for the health monitoring of mice in breeding and experimental units^13^, were found in mice with reduced PTS. We then set out to identify this *Bordetella* species by next generation sequencing. The structural and functional impact on the lung and trachea was analysed by light and electron microscopy, by BAL analysis and measurements of cilia-driven transport and beat frequency in *Bordetella* carriers versus non-carriers. Once isolated and identified as *B. pseudohinzii*, we used explanted tracheas to identify the cellular target of this species. Our data demonstrate that *B. pseudohinzii* can occur at high incidence in mouse colonies kept according to current FELASA recommendations, despite displaying little to none overt clinical symptoms. Affected mice present extensive airway inflammation with epithelial remodelling and functional impairment of clearance mechanisms. Therefore, we feel it justified to recommend regular testing for this *Bordetella* species and aiming to exclude it from facilities that provide mice for experimental lung research. On the other hand, controlled colonization and infection with *B. pseudohinzii* may serve as a model to investigate mechanisms of mucociliary clearance.

## Results

### Microbiology

**Figure 1:**
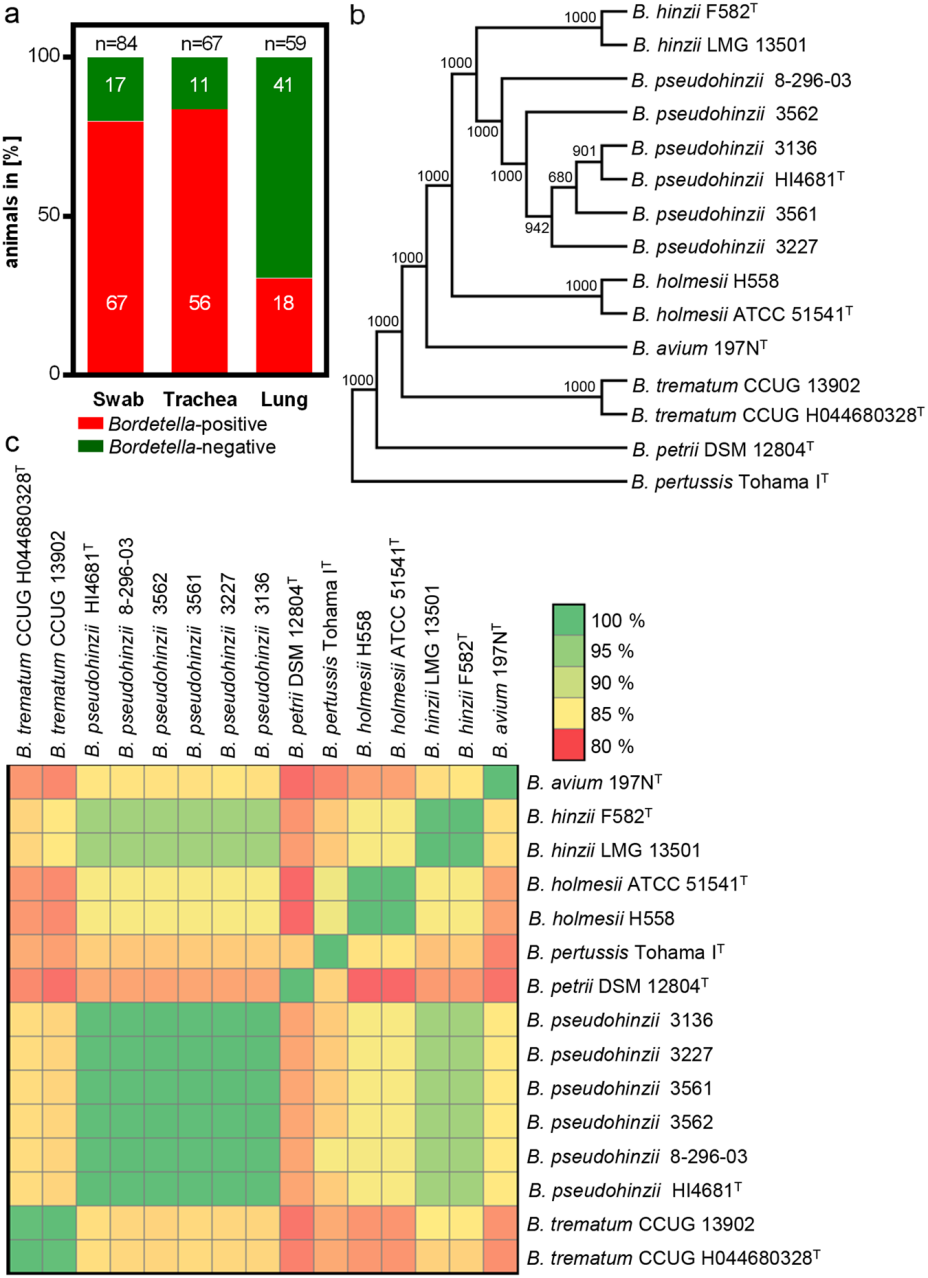
Occurrence of *B. pseudohinzii* in a laboratory mouse colony. (**a**) Prevalence of *B. pseudohinzii* in the oral cavity (swab), trachea and lung of mice confirmed by MALDI-TOF MS analysis and subsequent next generation genome sequencing of bacterial isolates. (**b**) Phylogenetic tree based on the amino acid sequences of 1649 core genes shared among 15 *Bordetella* genomes. The tree was constructed using the neighbour-joining method. Boot strap values are indicated. (**c**) Heatmap of the average nucleotide identity shared between different *Bordetella* species.

A high prevalence of disturbed tracheal PTS emerged in September 2016, and we began to subject samples of all animals used for experiments addressing pulmonary structure and function to microbiological diagnosis. Utilizing matrix-assisted laser desorption/ionization time-of-flight mass spectrometry (MALDI-TOF MS), a high incidence of *B. hinzii* was detected. In the period between October 2016 and March 2017, 84 animals were analysed in total, 78 (93%) of which were *Bordetella*-positive, with high incidence in the pharynx (oropharyngeal swab; 80%), trachea (84%), and lungs (30%; Fig. 1a).

In view of the fact that this MALDI-TOF MS (database by BRUKER) approach does not allow for a discrimination between *B. hinzii* and the very recently discovered and closely related species *B. pseudohinzii*^7,8^, the genomes of four isolates (strains 3136, 3227, 3561 and 3562) were sequenced using Illumina’s NextSeq. 500. In brief, the mean Phred quality score per sequence read was Q > 32 after adapter trimming and quality clipping, the number of contigs per strain ranged between 60–77 and the N50 coverage varied between 29–78 among the sequenced genomes (Supplementary Tables 2 and 3). Reference based SNP analysis indicated that all four isolates were highly related to *B. pseudohinzii* 8–296–03 (average 106.5 SNPs) compared to *B. hinzii* LMG 13501 (average 72546.75 SNPs) (Supplementary Fig. 1a). Phylogenetic analysis by EDGAR^16^ based on the amino acid sequences of 1649 core genes shared among 15 *Bordetella* genomes clustered the isolated strains to the *B. pseudohinzii* branch (Fig. 1b), which was also confirmed by ANI (Fig. 1c). Further characteristic features allowing discrimination from *B. hinzii* were the presence of the type II-C CRISPR Cas system reported in *B. pseudohinzii*^7^ and the absence of the typical D-galactonate utilization gene region present in *B. hinzii* but not in *B. pseudohinzii*^8^ (illustrated by GECO^17^, Supplementary Fig. 1b,c). Also, phylogenetic analysis of 16S rRNA gene sequences confirmed identity to *B. pseudohinzii* (Supplementary Fig. 2).

**Figure 2:**
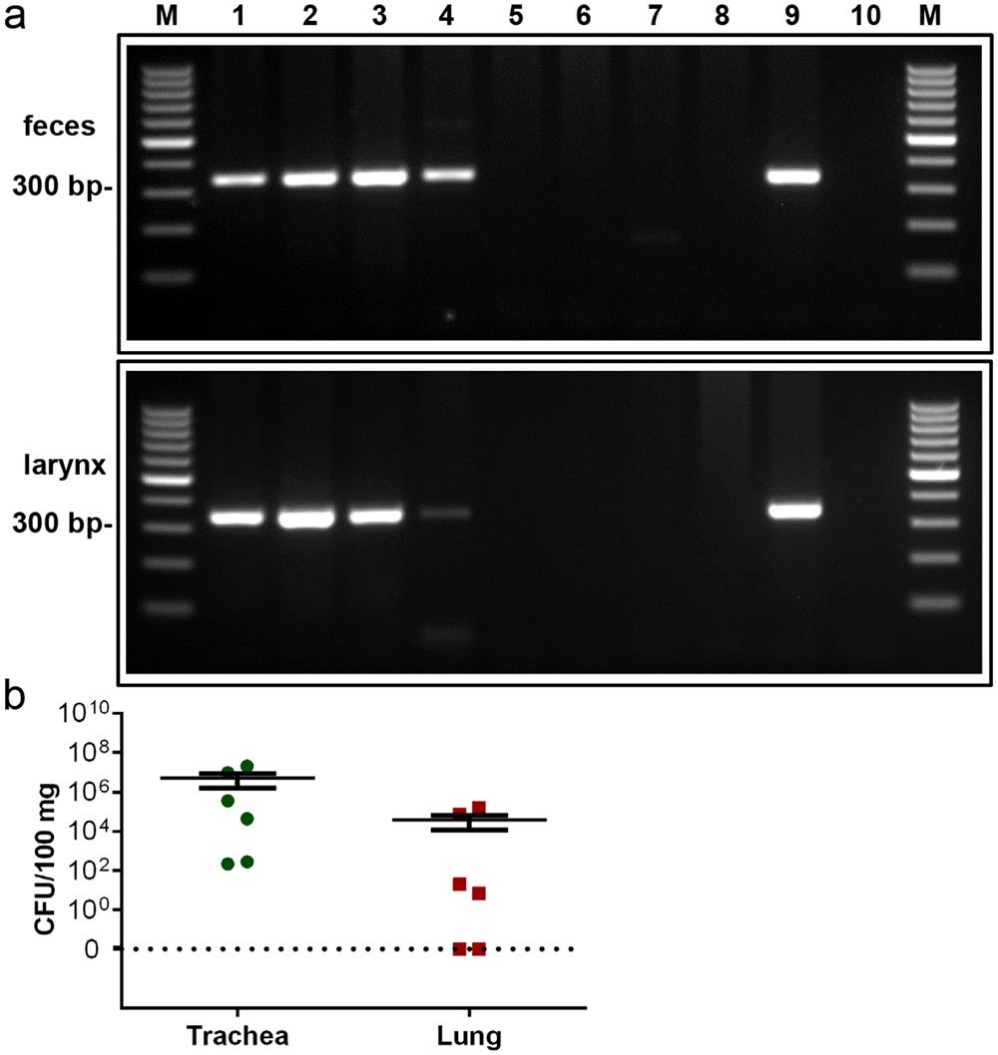
Detection of genomic *Bordetella* DNA by PCR. (**a**) A PCR product of 318 bp was amplified with *B. pseudohinzii*-specific primers from DNA of faeces (upper panel) and larynx (lower panel) from infected (lanes 1–4) and non-infected (lanes 5–8) mice, as classified by MALDI-TOF MS. DNA isolated from cultured *B. pseudohinzii* (strain 3227) served as a positive control (lane 9). Control runs without template were negative (lane 10). M: 100 bp molecular weight marker. (**b**) Trachea and lung taken were taken from 6 animals which were positive for *B. pseudohinzii* by PCR of faecal pellets, homogenized, and plated on selective agar plates. Number of colony forming units (CFU) is given per 100 mg of tissue. Individual data points shown; horizontal bar and whiskers indicate mean and standard error of the mean. Weight of tissue samples and CFU/organ are provided in Supplementary Table 1.

Accordingly, genomic *B. pseudohinzii* DNA was identified by PCR in faecal and laryngeal samples from animals in which *B. hinzii* was diagnosed in the trachea by MALDI-TOF MS (Fig. 2a). Samples tested negative by MALDI-TOF MS were also *Bordetella*-negative in PCR (Fig. 2a). The PCR assay targets a 318 bp-region of the outer membrane protein A (*ompA*) gene of *B. pseudohinzii*. Nucleic acid sequencing of the PCR-product showed 100% identity to the *ompA* sequence of *B. pseudohinzii* (GenBank accession number CP016440) and 98%-99% homogeneity to available *B. hinzii* sequences (GenBank accession numbers CP012076, CP012077, AM748263, AM478264, AM478265, LT906461).

From 6 animals with *B. pseudohinzii*-positive faecal PCR, CFU values were determined in homogenized trachea and lung. None of the animals used for this assay was positive for *Pasteurella pneumotropica*. Colonies from the agar plates were subjected to MALDI-TOF MS and diagnosed as *B. hinzii*, confirming that CFU counts indeed reflected *Bordetella* and that MALDI-TOF MS does not discriminate between *B. pseudohinzii* and *B. hinzii*. Colonies were obtained from all tracheas, and CFU ranged from 2.2 × 10^2^ to 2 × 10^7^/100 mg tissue, and from 5.6 × 10^1^ to 4.9 × 10^6^ per entire organ (Fig. 2b, Supplementary Table 1). From two of the lungs, no bacteria were retrieved, and the values in the other 4 ranged from 0.7 × 10^1^ to 1.5 × 10^5^ CFU/100 mg tissue and from 1.3 × 10^1^ to 1.9 × 10^5^ CFU/lung (Fig. 2b, Supplementary Table 1).

In transmission electron microscopy, fully sequenced *B. pseudohinzii* presented as rod-shaped coccobacillus (Supplementary Fig. 3), 1,550 ± 66 nm (mean ± SEM; n = 20) in length, with peritrichous flagellae of 2,483 ± 133 nm (n = 42), as also previously described by Ivanov and coworkers^8^.

### Histopathology and bronchoalveolar lavage

**Figure 3:**
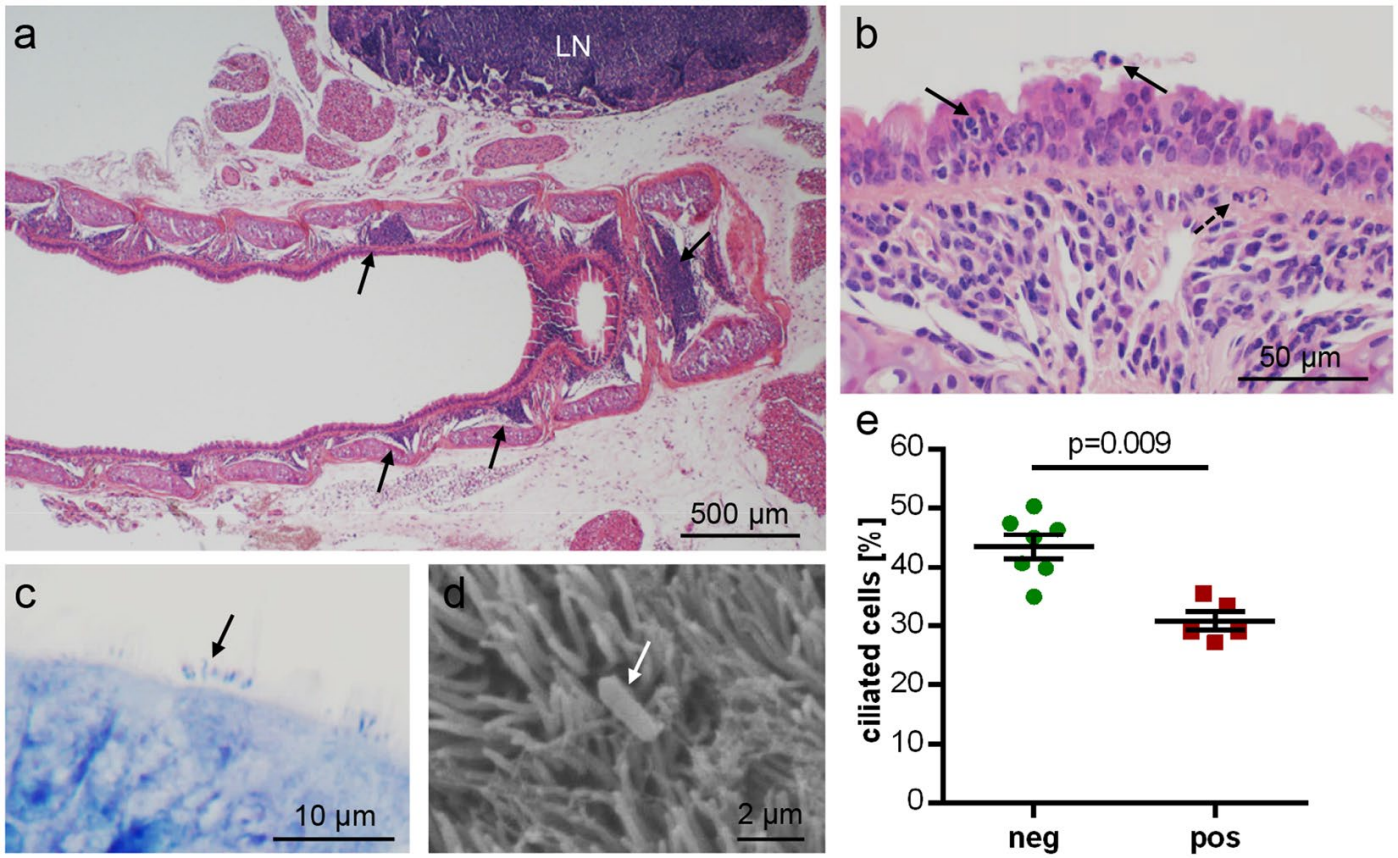
Tracheal histopathology in *B. pseudohinzii*-infected mice; micrographs are taken from animals which were negative for *Pasteurella pneumotropica*. (**a**,**b**) H&E-stained paraffin section. (**a**) *Arrows* point at focal infiltrations in the lamina propria in a longitudinal section. LN = adjacent lymph node with lymphoid hyperplasia. (**b**) Neutrophilic granulocytes (*arrows*) have invaded and penetrated the epithelial layer. *Broken arrow* indicates neutrophil accumulation underneath the basal lamina. (**c**,**d**) Rod-shaped bacteria (*arrows*) are attached to kinocilia, as seen in a Giemsa-stained paraffin section (**c**) and in scanning electron microscopy (**d**). (**e**) The relative frequency of ciliated cells among epithelial cells, as evaluated in H&E-stained paraffin sections, is significantly reduced in *B. pseudohinzii*-positive (pos; n = 5) compared to *B. pseudohinzii*-negative mice (neg; n = 7). Individual data points shown; horizontal bar and whiskers indicate mean and standard error of the mean (Student’s unpaired t-test).

Histopathological changes were noted in the tracheal mucosa and in the lungs. The lamina propria of the trachea had multifocal to confluent infiltrations with neutrophils, lymphocytes and plasma cells. Neutrophils in particular also entered the epithelial layer and the tracheal lumen (Fig. 3a,b).

Rod-shaped bacteria were seen at the surface of ciliated epithelial cells in Giemsa-stained sections (Fig. 3c). Scanning electron microscopy revealed single rod-shaped bacteria attached to kinocilia of ciliated cells (Fig. 3d). The relative frequency of tracheal ciliated epithelial cells was lower in *Bordetella*–positive animals compared to *Bordetella*-negative animals (30.9 ± 1.5%, n = 5, and 43.5 ± 5.3%, n = 7, respectively; *p* = 0.0009; Fig. 3e). None of the animals used for these cell counts was positive for *Pasteurella pneumotropica*.

**Figure 4:**
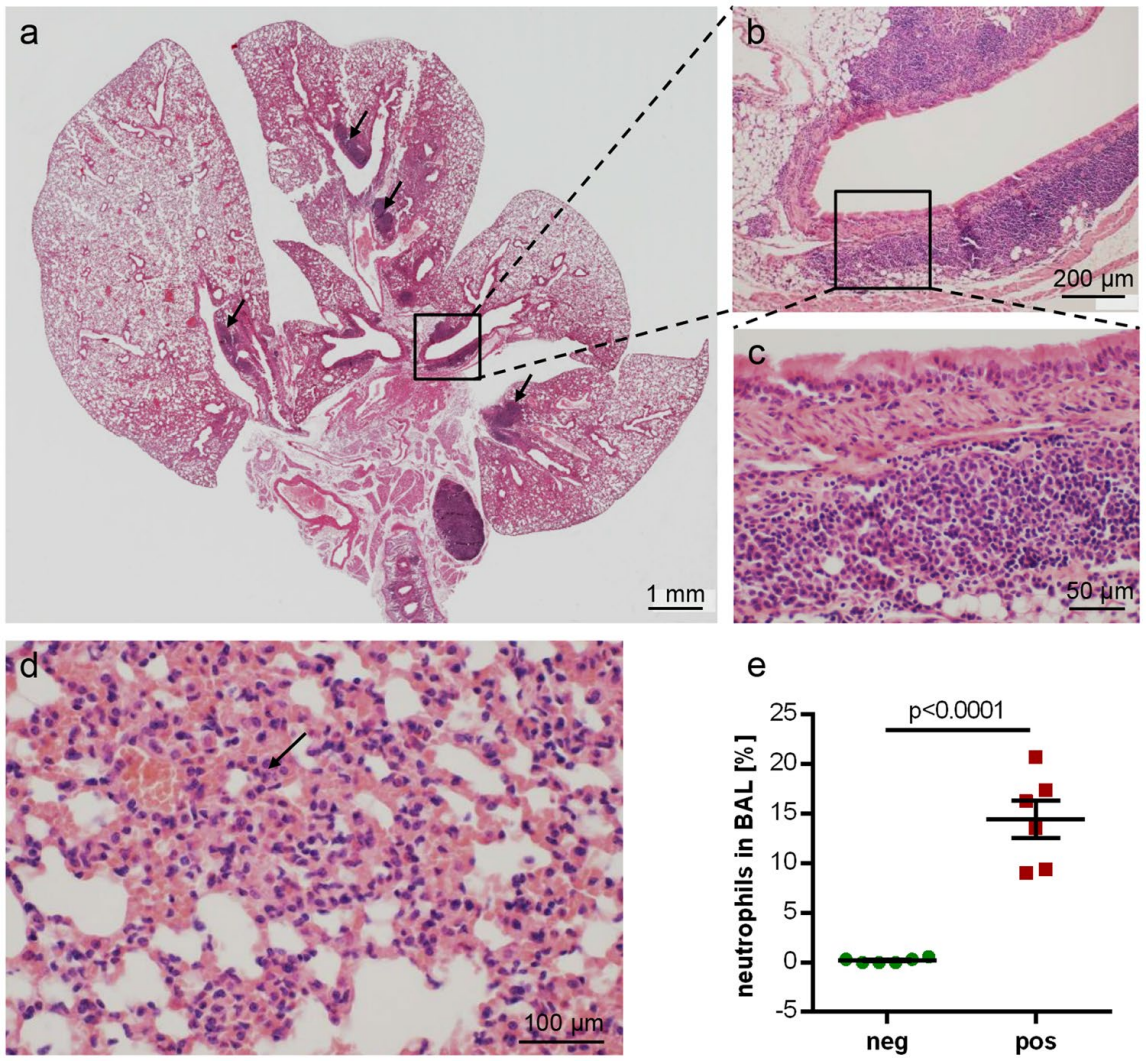
Lung histopathology in *B. pseudohinzii*-infected mice. (**a–d**) H&E-stained paraffin section; (**c**) and (**b**) represent higher magnifications of boxed areas in (**b**) and (**a**), respectively, demonstrating BALT aggregates; additional BALT is indicated by arrows in (**a**). The lung section depicted in **a–c** is taken from an animal which was also positive for *Pasteurella pneumotropica*. (**d**) Interstitial pneumonia with neutrophils (*arrow*). (**e**) The incidence of neutrophils is significantly elevated in the BAL fluid from *B. pseudohinzii*-positive (pos; n = 6) compared to *B. pseudohinzii*-negative mice (neg; n = 6) (mean ± SEM; Student’s unpaired t-test).

The lungs presented with multifocal bronchointerstitial pneumonia with dominating neutrophils and macrophages in major airways and alveolar spaces (Fig. 4e). Accordingly, neutrophils made up between 9% and 21% (mean: 14%; n = 6; Fig. 4d) of cells in the BAL fluid from *Bordetella*-positive animals, whereas they accounted for less than 0.6% throughout in *Bordetella*-negative animals (mean: 0.2%; n = 6). None of the animals used for cell counts in BAL was positive for *Pasteurella pneumotropica*, one was positive for *Klebsiella oxytoca* (this was the only *Klebsiella*-positive animal identified in the entire course of the study). Lymphocytes, plasma cells and macrophages formed peribronchial cuffs. Prominent aggregates of bronchus-associated lymphoid tissue (BALT), not seen in *Bordetella*-negative animals, were present close to the main bronchi in the hilar regions (Fig. 4a,b and c). Furthermore, a lymphoid hyperplasia of lymph nodes could be observed (Fig. 3a). Three of the 6 *Bordetella*–positive animals used for histopathological assessment were also positive for *Pasteurella pneumotropica*. There were no criteria identified that distinguished these animals from those carrying only *B. pseudohinzii*. Specifically, BALT formation was seen in both *Pasteurella*-positive (Fig. 4a–c) and *Pasteurella*-negative (Fig. 3a) samples.

### *In vitro*-infection of tracheal explants

**Figure 5:**
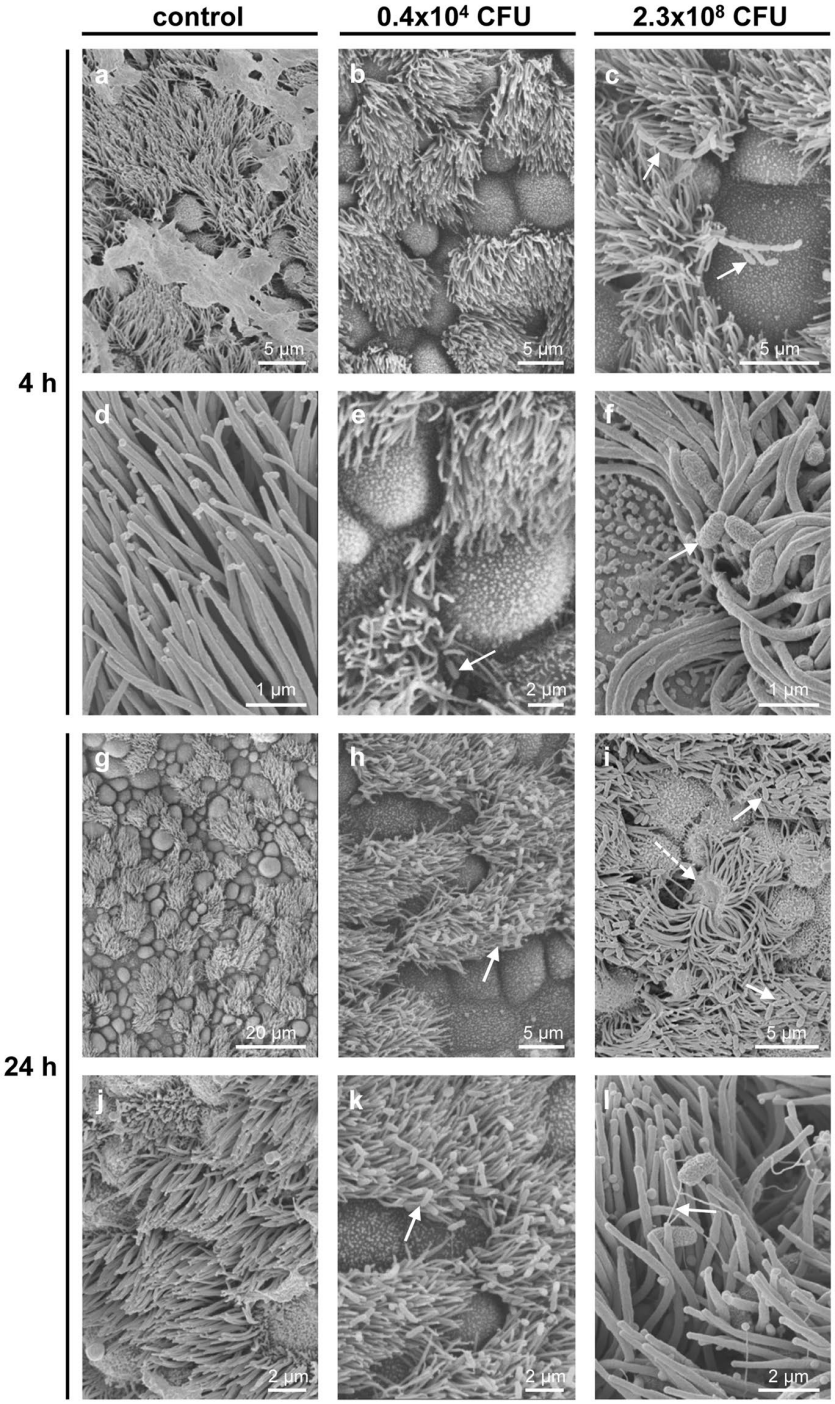
*B. pseudohinzii* (strain 3227) attaches to cilia and damages the epithelium in an *in vitro*-infection model. Tracheal explants were cultured for 24 h, with following addition of bacteria for 4 h or 24 h, and analysed by scanning electron microscopy. (**a**,**d**) Upon cultivation for 24 + 4 h without adding of bacteria, cilia are regularly orientated and no bacteria are visible. (**b**,**e**) The trachea was cultivation for 4 h in 1 ml medium with 0.4 × 10^4^ CFU *B. pseudohinzii*. A single bacterium is seen attached to cilia (*arrow*). (**c**,**f**) Cultivation for 4 h with a higher dose of *B. pseudohinzii* (2.3 × 10^8^ CFU). No obvious alterations of cilia or ciliated cells can be observed. Bacterial chains are attached to cilia (*arrows*). (**g**,**j**) Cultivation for 48 h without adding of bacteria. Ciliated cells are intact and cilia show no signs of damage. (**h**,**k**) Cultivation for 24 h with 0.4 × 10^4^ CFU *B. pseudohinzii*. (**i**,**l**) Cultivation for 24 h with a higher dose of *B. pseudohinzii* (2.3 × 10^8^ CFU). Increased numbers of bacteria are attached to cilia (*arrows*), cilia look flattened. Single ciliated cells are massively damaged (*dotted arrow*).

**Figure 6:**
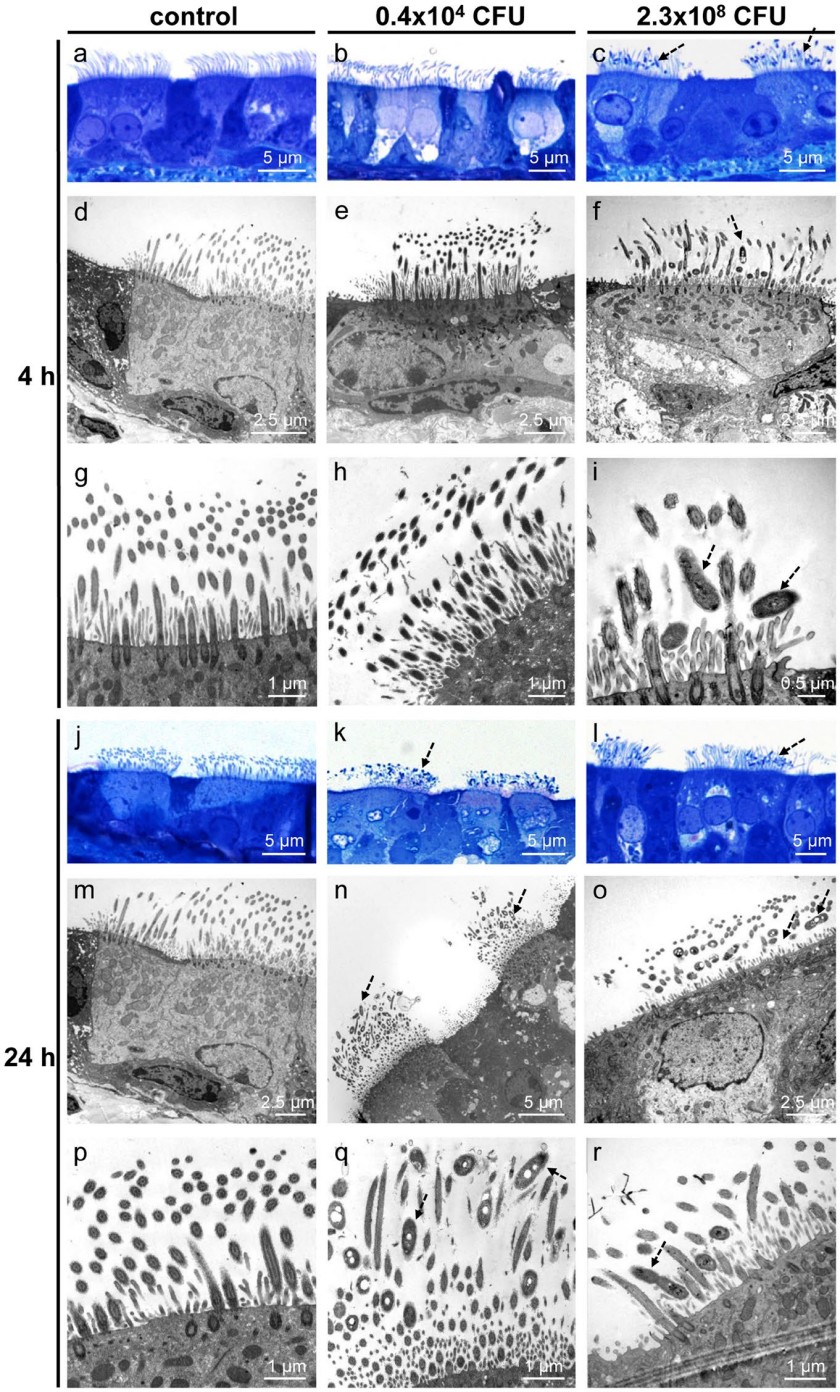
*B. pseudohinzii* (strain 3227) attaches to and intermingles with cilia in an *in vitro*-infection model. Tracheal explants were cultured for 24 h, with following addition of bacteria for 4 h (**a–i**) or 24 h (**j–r**). (**a–c**) Semithin sections of tracheal explants cultured for 24 + 4 h, light microscopy. (**a**) Without adding of bacteria, cilia are normally orientated and no bacteria are visible. (**b**) *B. pseudohinzii* (0.4 × 10^4^ CFU) was added for 4 h. No bacteria are seen. (**c**) *B. pseudohinzii* (2.3 × 10^8^ CFU) was added for 4 h. Bacteria attach to cilia (*dotted arrows*). (**d–i**) Transmission electron microscopy. (**d**,**g**) Control experiment. Tracheas were cultured for 24 + 4 h without bacteria. Cilia appear normal in length and orientation. No signs of cell damage. (**e**,**h**) No obvious difference to controls 4 h after incubation with 0.4 × 10^4^ CFU *B. pseudohinzii*. (**f**,**i**) *B. pseudohinzii* (2.3 × 10^8^ CFU) was added for 4 h. Bacteria are located between the cilia (*dotted arrow*), (**j**,**m**,**p**) Control. Explants cultivated for 24 h without bacteria demonstrate unaltered ciliated cells in light (**j**) and transmission electron microscopy (**m**,**p**). (**k**,**n**,**q**). *B. pseudohinzii* (0.4 × 10^4^ CFU) was added for 24 h. Bacteria (*dotted arrows*) intermingle with kinocilia. They are not seen at non-ciliated cells. (**l**,**o**,**r**) *B. pseudohinzii* (2.3 × 10^8^ CFU) was added for 24 h. The number of bacteria (*dotted arrows*) is increased, cells with shortened of cilia can be observed in transmission electron microscopy (**o**,**r**).

Ciliated cells maintained intact cilia and normal ciliary beat frequency (CBF) in non-infected tracheal explants for up to 52 h, the longest time point examined (Supplementary Fig. 4). In these controls, bacteria were detected neither in semithin sections nor by scanning or transmission electron microscopy (Figs 5a,d,g and j and 6a,d,g,j,m and p). *B. pseudohinzii* was added to explanted tracheas at 5 different doses for 4 h and 24 h, respectively. With respect to CFU/entire trachea, the 3 lower doses were in the range of what we had recovered from infected animals. After 4 h, no bacteria were detected at the lowest dose, and only very few, single rod-shaped bacteria adhered to cilia at the two next higher doses (Figs 5b,e and 6b,e and h; Supplementary Fig. 5a,c; Fig. 6a,b,d,e,g and h). At a dose of 2.3 × 10^8^ CFU/ml, bacteria regularly adhered to and intermingled with cilia (Fig. 6c,f and i), and bacterial chains, indicative of replication, were attached to cilia (Fig. 5c,f). At the highest dose (4.6 × 10^9^ CFU/ml), microcolonies were observed at ciliated cells, cilia were shortened, distorted, and irregularly arranged (Supplementary Fig. 5d; Fig. 6c,f and i). The surface of non-ciliated cells was devoid of bacteria at all doses (Figs 5a–f and 6a–i).

At 24 h of incubation, bacteria attached to ciliated cells in all conditions except controls with a CFU-dependent increase in numbers (Figs 5g–l; 6j–r; Supplementary Fig. 5e–h; Fig. 6j–r). At the highest dose, only remnants of cilia remained and extensive blebbing of apical cell poles of ciliated and non-ciliated cells was observed (Supplementary Fig. 5f,h).

### Cilia-driven transport and beat frequency

**Figure 7:**
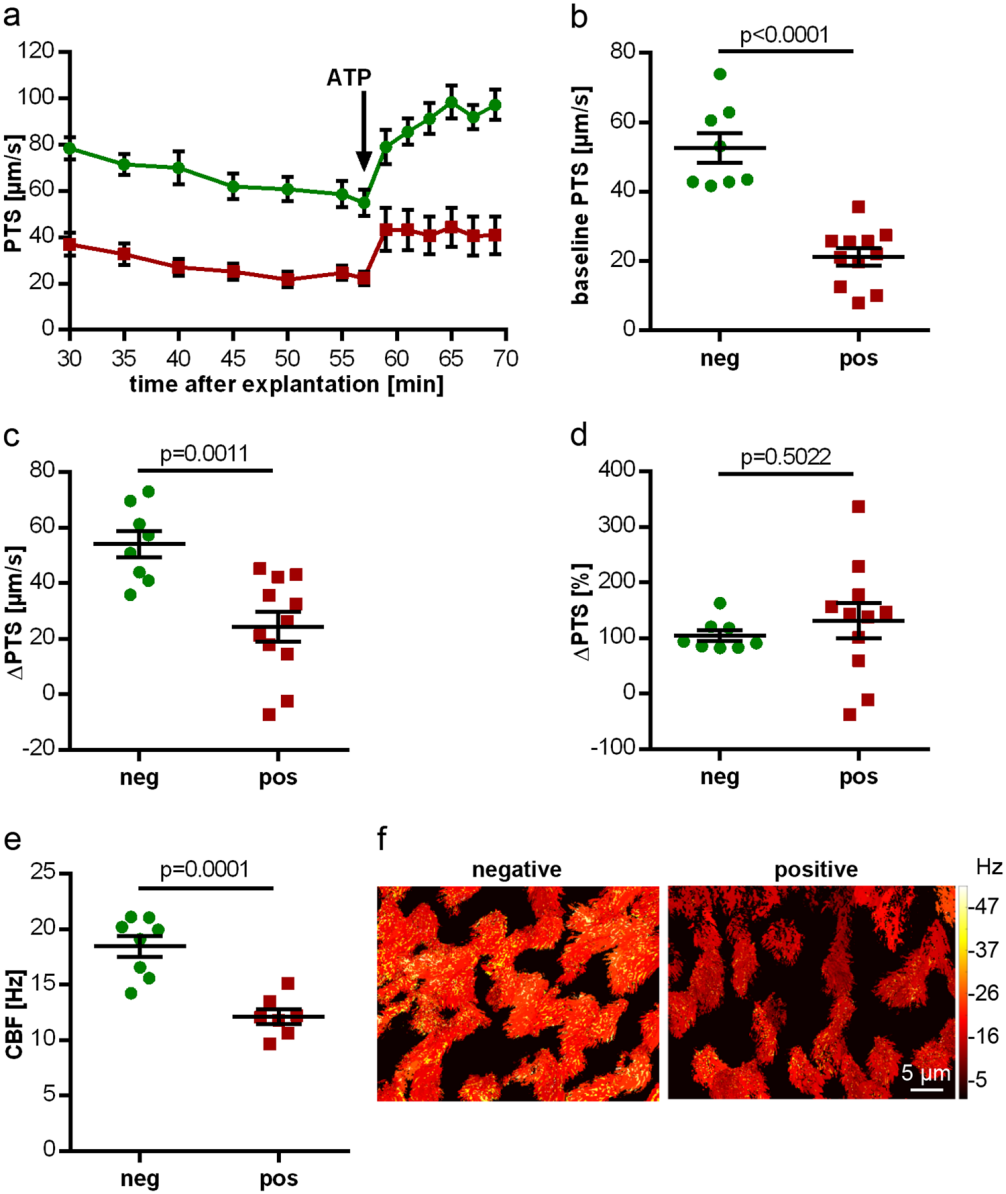
Altered PTS and CBF in *B. pseudohinzii-*positive animals. (**a**) PTS measurements of mice tested positive (red; n = 11) and negative (green; n = 8) (mean ± SEM) for *B. pseudohinzii*. In both groups ATP leads to an increase in PTS, but baseline PTS is reduced in *B. pseudohinzi*-positive animals. (**b–d**) Statistical analysis of the experimental setup shown in (**a)**. Individual data points with mean ± SEM are shown. (**b**) Baseline PTS of *B. pseudohinzii-*positive animals is significantly lower compared to negatively tested animals. (**c**) Absolute increase in PTS after 8 min of ATP application is significantly lower in *B. pseudohinzii*-positive animals. (**d**) ATP-driven increase, expressed in percentage relative to baseline, is not different between the groups. (**e**) Baseline CBF of *B. pseudohinzii*-positive animals is significantly decreased 27 min after explantation of the trachea. Individual data points and mean ± SEM are shown. *B. pseudohinzii*-positive animals (red; n = 7) and *B. pseudohinzii-*negative animals (green; n = 8). The microscopic images show the mucosal surface with false colour coding of CBF of single ciliated cells; brighter colours represent higher CBF. Decrease in CBF in *B. pseudohinzii*-positive animals is a general feature of the majority of ciliated cells and is not restricted to single ciliated cells. All p-values were calculated by Student’s unpaired t-test.

Baseline PTS was significantly lower in explanted tracheas taken from *Bordetella*-positive compared to *Bordetella*-negative animals (21.2 ± 8.3 µm/s, n = 11, and 52.7 ± 4.3 µm/s, n = 8, respectively; p < 0.0001; Fig. 7a,b). ATP-induced increase in PTS, recorded as absolute values of micrometer per second, was smaller in *Bordetella*-positive than in *Bordetella*-negative animals (24.4 ± 5.4 and 53.9 ± 4.8 µm/s; p = 0.0011; Fig. 7c). Expressed in percent of baseline value before ATP-stimulation, however, no significant difference was noted (104.7 ± 9.8% versus 130.8 ± 31.5%; p = 0.5022; Fig. 7d). Among the 11 *Bordetella*-positive animals analysed in this assay, 4 were also positive for *Pasteurella pneumotropica*. This had no additional negative impact on PTS, as it was even slightly higher (33.4 ± 3.3 µm/s) in tracheas taken from these animals than in those from *Bordetella*-positive/*Pasteurella*-negative animals (19.5 ± 3.3 µm/s).

Baseline CBF was also significantly reduced in *Bordetella*-positive animals (12.1 ± 0.7 versus 18.5 ± 1.1 Hz; n = 7 each; p = 0.0001; Fig. 7e). Among the 7 *Bordetella*-positive animals analysed in this assay, 4 were also positive for *Pasteurella pneumotropica*. This did not correlate with differences in CBF (*Pasteurella*-positive: 12.7 ± 1.0 Hz, n = 4; *Pasteurella*-negative: 11.3 ± 0.8 Hz, n = 3).

**Figure 8:**
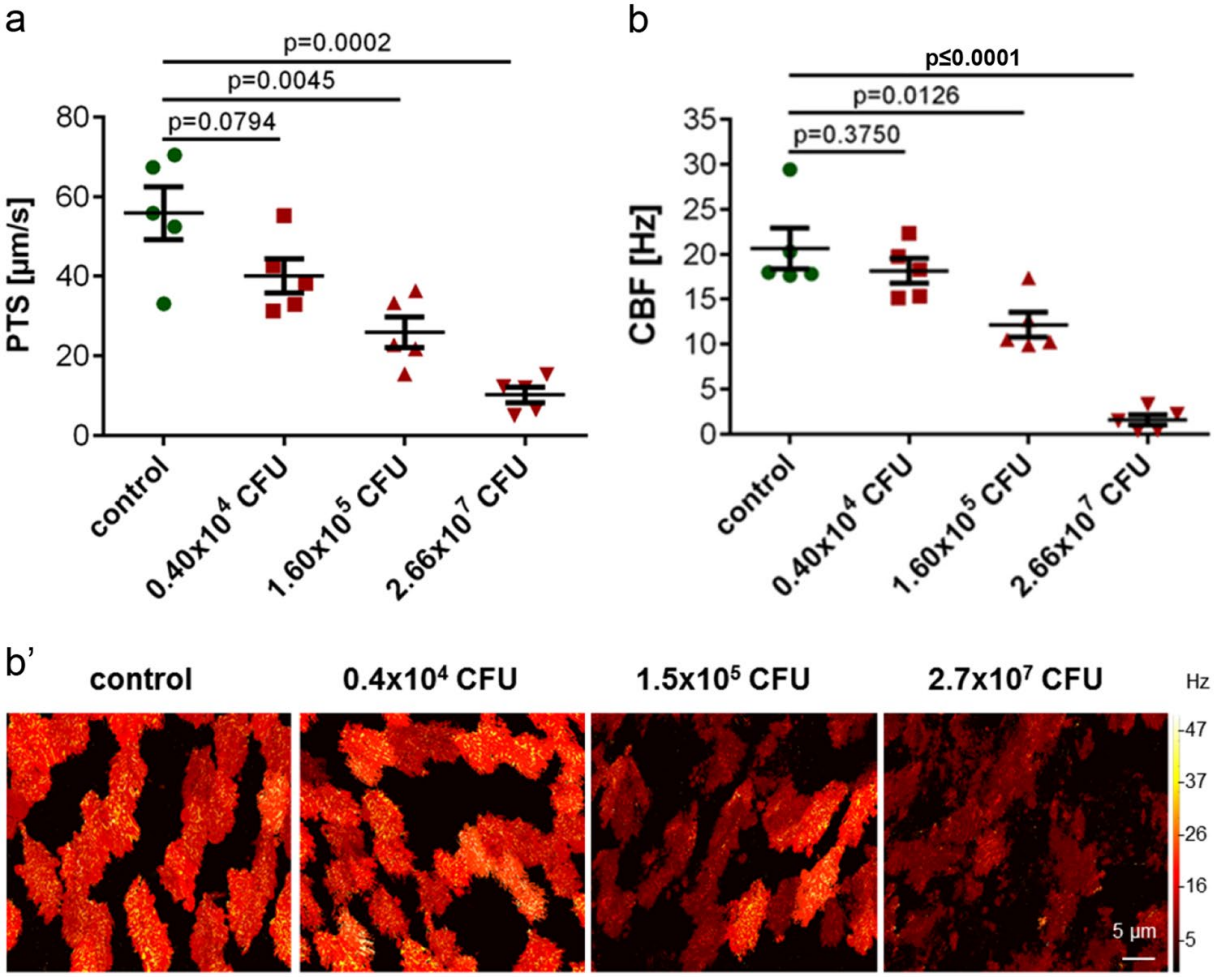
Altered baseline PTS and CBF of tracheal rings cultured *in vitro* for 4 h with *B. pseudohinzii* (strain 3227). Baseline PTS (**a**) and baseline CBF (**b**) of tracheal explants incubated with 1.60 × 10^5^ and 2.66 × 10^7^ CFU (red) are significantly decreased compared to the corresponding control without bacteria (green); n = 5 in each group; (mean ± SEM; Student’s unpaired t-test). The microscopic images in b’ show the mucosal surface with false colour coding of CBF of single ciliated cells; brighter colours represent higher CBF. Decrease in CBF with increasing *B. pseudohinzii* numbers is a general feature of the majority of ciliated cells and is not restricted to single ciliated cells.

Next, the acute effects of *Bordetella pseudohinzii* on cilia-driven transport and beat frequency were investigated in explanted tracheas from *Bordetella*-negative animals exposed for 4 h to 3 different concentrations of bacteria. With respect to CFU/entire trachea, the 2 lower doses were in the range of what we had recovered from infected animals, and the highest dose was one order of magnitude higher. In control explants kept for 4 h without bacteria, baseline PTS (55.8 ± 3.7 µm/s) and CBF (20.6 ± 2.5 Hz) did not differ significantly from values obtained from freshly used tracheas. In both readouts, a CFU-dependent decline of function was observed with about 50% reduction at 1.6 × 10^5^ CFU and practically complete loss of ciliary function and particle transport at 2.66 × 10^7^ CFU (Fig. 8). In the videos taken for analysis of these parameters, attachment of bacteria to cilia was seen in a CFU-dependent manner, beginning at 0.4 × 10^4^ CFU.

## Discussion

Until now, isolates of the recently identified non-classical *Bordetella* species *B. pseudohinzii* that were unequivocally identified by whole genome sequencing had been obtained from mice in various facilities in the United States^7,8,18^ and Malaysia^10^. In addition, there have been reports on the widespread occurrence of *B. hinzii* in mice in experimental facilities in Japan^11,12^, which, based upon the published partial sequences, most likely have monitored *B. pseudohinzii* instead^8,9^. In Europe, so far only one case has been reported. One mouse imported from Australia to the University of Düsseldorf in Germany had first been diagnosed as being infected with *B. hinzii*, and subsequent resequencing of this strain identified it as *B. pseudohinzii*^8,19^. In our study we obtained 4 isolates of *B. pseudohinzii* from laboratory mice bred and kept at another German SPF facility, showing that the occurrence of this species is not restricted to East Asia, Australia and North America.

At least in the colonies used for experimental work of our group, the incidence of *B. pseudohinzii* was high over a period of 6 months (93% of all animals tested; n = 84), which is far above the incidence reported for *B. hinzii*, probably representing *B. pseudohinzii* as discussed above^8,9^, in experimental facilities in Japan^12^. There, 1.6% (195 out of 12,192) of all tested mice were positive, and 2.8% of facilities (44 out of 1572) in universities and research institutes were affected. Notably, no *B. hinzii-*positive mice were found in 127 pharmaceutical companies surveyed. Taking only those facilities with positive findings, 32% of mice were positive (195 out of 613)^12^. While this is still less than half of the frequency we have observed, it has to be considered that a similarly high prevalence might have occurred in single facilities which cannot be extracted from the pooled data. Most importantly, the numbers on prevalence in Japan are derived from a systematic study testing regardless of clinical or experimental evidence for pulmonary problems, whereas our analysis was biased, because it was triggered by findings of impaired mucociliary clearance. Similar to our counts, 12 out of 15 animals (representing 7 mouse strains) taken from a facility where analysis was initiated due to high neutrophil counts in BAL fluid, were positive for *B. pseudohinzii* in faecal PCR^9^. Altogether, it appears that spread of *B. pseudohinzii* in mouse facilities may be focal but not regionally restricted, and can reach peaks of prevalence of > 80% in mice with C57BL/6 J genetic background, as reported here.

Colonization with *B. pseudohinzii* may predispose for infection with other lung pathogens, which may render interpretation of data difficult. Thus, samples from all animals used for histopathology or functional readouts were analysed by MALDI-TOF MS. Among potential candidates, *Klebsiella oxytoca* was identified in only one animal, so that it could be neglected in this context. *Pasteurella pneumotropica* was either entirely absent from cohorts used in a certain assays, or no significant differences between animals additionally carrying this bacterium and those being positive for *B. pseudohinzii* alone were noted. In line with this observation, experimental inoculation with *Pasteurella pneumotropica* caused histopathological pulmonary changes only in immunodeficient (NOD/ShiJic-*scid*/Jcl), but not in immunocompetent Crlj:CD1 (ICR) mice in another study^20^. Other potential lung pathogens were not identified in our study. Thus, we ascribe the histopathological and functional changes that we have observed to *B. pseudohinzii*.

Pulmonary histopathology and cellular composition of BAL fluid have been previously reported for mice with *B. pseudohinzii*-positive faecal PCR, with elevated neutrophils in the BAL fluid up to 20% in 10/12 animals and peribronchiolar infiltrates exceeding that seen in negative animals in 5/8 cases^9^. This is principally consistent with our current findings, although signs of inflammation were generally stronger in our cohort and we observed no overlap with negative animals in that none of the positive animals had neutrophil counts lower than 8%. Also, pulmonary histopathology was not restricted to peribronchiolar infiltrates but presented as BALT formation and bronchointerstitial pneumonia. Similarly, bronchopneumonia with an increase in lymphoid tissue has been reported for mice taken from Japanese facilities, at that time classified as carrying *B. hinzii*^11,12^, now reconsidered as *B. pseudohinzii*^8,9^.

Most *Bordetella* species affecting the respiratory tract, however, do not primarily target the peripheral lung but ciliated cells of the airway epithelium (e.g. *B. pertussis*^21^, *B. bronchoseptica*^22^, *B. avium*^23^). Our data on explanted mouse tracheal rings demonstrate that kinocilia are also the primary site of attachment of *B. pseudohinzii*, with formation of bacterial chains indicative of replication. At bacterial CFU numbers corresponding to those which we determined from tracheas of infected mice, attachment to kinocilia was seen as early as 4 h of incubation. This was more pronounced in live videos taken in the course of analysis of ciliary function than in samples processed for electron microscopy, which may be due to the numerous washing steps included in the latter procedure. Dependent on time of incubation and bacterial load, extensive damage and loss of ciliated cells occurred, consistent with the observation of a reduced frequency of ciliated cells in the tracheas of *B. pseudohinzii-*positive mice.

Importantly, not only the number of ciliated cells was decreased in *B. pseudohinzii-*positive mice, but those remaining were functionally impaired so that cilia beat slower. Collectively, this resulted in more than 50% reduction in cilia-driven PTS, a readout parameter for mucociliary clearance. Our experiments on isolated tracheas from *B. pseudohinzii-*negative mice exposed for 4 h to this bacterium in culture revealed similar reduction in PTS and CBF, so that direct effects on ciliated cells appear to contribute more to the overall effect than epithelium remodelling.

Mucociliary clearance is a primary innate defence mechanism that eliminates inhaled particles, including bacteria, from the airways. It is driven by the coordinated beating of cilia that generate a vectorial fluid transport and propel the surface liquid layer consisting of a periciliary fluid and mucus towards the larynx^24,25^. Viscosity of the airway surface liquid, mainly determined by mucins, is another independent determinant of overall efficacy of mucociliary clearance. Our data were generated in mucus-free explanted tracheas, where solely the cilia-driven component is assessed^15,26,27^. While this offers the advantage to analyse in detail the mechanisms underlying the cilia-driven component, the PTS measured therein is not necessarily in direct linear relation to overall mucus or bacterial clearance from the trachea. Still, defects in ciliary function generally do have a negative effect on overall clearance, as it is exemplified in various forms of inherited ciliopathies^28^.

The closest relative of *B. pseudohinzii*, i.e. *B. hinzii*, has been isolated from respiratory secretions of cystic fibrosis patients which had impaired mucociliary clearance because of increased mucus viscosity due to their underlying disease^29,30^. Data on direct ciliotropic effects of *B. hinzii*, however, have yet not been reported in affected patients or in the natural hosts. However, the closely related species *B. avium* also attaches to kinocilia in explanted turkey tracheal rings and causes apoptosis of ciliated cells^31^. Intranasal inoculation of *B. avium* leads to extensive tracheitis with loss of ciliated cells^2,32^ and, again, this is linked to impaired mucociliary clearance. Not in the early phase, but at 21 days after inoculation, overall clearance of inhaled ^99m^technetium-sulfur colloids was significantly reduced as assessed by scintigraphy^33^, indicating that epithelium remodelling rather than acute toxic effects is the underlying cause in this condition.

Despite the extensive airway inflammation with epithelial remodelling and a drastic functional impairment of an important clearance mechanism, the affected animals had no overt clinical symptoms in our facility. Microbiological diagnosis was initiated because of suspicious functional data sets rather than by compromised general condition of the animals. This is in accordance with a recent report from a facility in the United States, where increased neutrophil counts in BAL fluid in otherwise inconspicuous mice triggered diagnosis of *B. pseudohinzii*, revealing a prevalence of 80% (12/15)^9^. The index case for isolation of *B. (pseudo)hinzii* from mice (a female C57BL/6 mouse), however, presented sneezing with a chattering sound, and these clinical signs together with mild dyspnea were observed in all Jcl:ICR mice from day 3 up to at least 28 days after nasal inoculation with this bacterial isolate^11^. Among inoculated immunocompromised mice (NOD/ShiJic-*scid*Jcl), 2 out of 10 were euthanised because of severe dyspnea and additional two died spontaneously in the experimental period (up to day 28) without prior signs of severe symptoms^11^. Thus, mice carrying this non-classical *Bordetella* strain present a broad range from subclinical colonization over modest respiratory infection to even lethal infection in immunocompromised animals.

In conclusion, our data demonstrate that *B. pseudohinzii* physically and functionally targets ciliated cells of the airway epithelium in C57BL/6 J mice. The affected animals present extensive airway inflammation with epithelial remodelling and functional impairment of clearance mechanisms, despite showing little to none overt clinical symptoms. Therefore, we feel it justified to recommend regular testing for this *Bordetella* species in animal facilities and aiming to exclude it from colonies that provide mice for experimental lung research. Otherwise, it may occur at high incidence in mouse colonies kept according to current FELASA recommendations. On the other hand, controlled colonization and infection with *B. pseudohinzii* may serve as a model to investigate mechanisms of compromised mucociliary clearance and microbial strategies to escape from this primary innate defence response.

## Materials and Methods

### Animals

All animals were housed under standard laboratory SPF conditions (10 h dark, 14 h light). In this facility, health monitoring of mice takes place quarterly following the FELASA recommendations for health monitoring of rodents^13^. Animals examined are aged stock animals. Serological monitoring via ELISA is performed for all viruses of the FELASA recommendations, and for *Clostridium piliforme* and *Mycoplasma pulmonis*. Examination for and differentiation of *Helicobacter* species is done via faecal PCR. Swab samples of trachea, caecum and vagina/prepuce are used for bacteriological cultivation and subsequent MALDI-TOF MS identification of cultivated agents. Fur, gross intestine and feces are examined microscopically for endo- and ectoparasites. Pathological examination of animals is performed yearly. By this monitoring the facility is identified as positive for Mouse norovirus, *Helicobacter* spp. (including *H. hepaticus*, *H. rodentium*, *H. typhlonius*), *Bordetella hinzii*, *Klebsiella oxytoca*, *Pasteurella pneumotropica*, *Staphylococcus aureus*, *Entamoeba muris*, Flagellates and *Tritrichomonas muris*.

All samples were taken after anaesthesia by isoflurane (5%) (Abbott, Wiesbaden, Germany) and killing of mice by cervical dislocation. Wild-type C57BL/6 J mice and five different transgenic mouse strains with C57BL/6 J background were tested for the incidence of *B. pseudohinzii* in our animal colonies.

For all functional experiments, wild-type C57BL/6 J mice of both sexes and at least 12 weeks of age were used. They were either bred in our animal facility or purchased from Janvier (St. Berthevin, France), and tested for *B. pseudohinzii* by bacteriological investigations including MALDI-TOF MS. Among the potentially pneumotropic bacteria listed above, *Klebsiella oxytoca* was identified in only one animal used for cell counts in BAL. Numbers of animals positive for *Pasteurella pneumotropica* are specified in detail for each experimental readout in the respective paragraph of the results chapter. All animals were held according to the European Community guidelines for the care and use of laboratory animals, and breeding and use of samples of sacrificed mice for further *in vitro*-experiments were registered by the responsible authorities at the Regierungspräsidium Giessen, Hesse, Germany.

### Bacteriological investigations

To examine bacterial colonization, the mice were dissected under sterile conditions and samples were taken from larynx, trachea and lung, around 20–40 mg each. In addition, samples from the pharynx were taken by wiping with a swab (Nerbe plus, Winsen, Germany).

Each sample was examined by streaking onto blood agar plate (Oxoid GmbH, Wesel, Germany), on brain heart infusion (BHI) agar (Oxoid GmbH) and selective agar plates (blood agar containing cephalexin hydrate [20 mg/ml; Sigma Aldrich]; nutrient broth no. 2 and agarose containing neomycin trisulphate salt hydrate [2 mg/ml; Sigma Aldrich] and bacitracin [3 mg/ml; Carl Roth, Karlsruhe, Germany]; water-blue metachrome-yellow lactose agar [acc. to Gassner, E. Merck]). For enrichment purposes, each sample was inoculated to nutrient broth no. 2 containing 10% bovine serum and broth were streaked onto blood and Gassner agar plate after 24 h incubation at 37 °C. Plates were examined after 24 h and 48 h, as well as for BHI agar after 72 h at 10% CO_2_. Morphologically diverse colonies were sub-cultivated. Pure cultures were identified using MALDI-TOF MS (Biotyper Version V3.3.1.0, Bruker Daltonics, Bremen, Germany) and the DB 5989 database.

To determine the total *B. pseudohinzii* CFU number per organ, tissue of animals tested positive for *B. pseudohinzii* by PCR was homogenized using a ball mill (MM 300; Retsch, Haan, Germany). The homogenate was then filtered using a cell strainer (100 µm pore size; Corning, Tewksbury, USA) and serial dilutions were plated onto selective agar plates (blood agar containing cephalexin hydrate [20 mg/ml; Sigma Aldrich]) and cultured for 48 h at 37 °C and 10% CO_2_. Bacterial colonies were counted and analyzed by MALDI-TOF MS.

### Genome sequencing and bioinformatics analysis

*Bordetella sp*. (strains 3136, 3227, 3561 and 3562) isolated from mice of the breeding facility in Giessen were genome sequenced using Illumina’s NextSeq. 500 next generation sequencing system. Chromosomal DNA was isolated using PureLink® genomic DNA mini kit (Life Technologies). Nextera XT paired-end libraries were sequenced using NextSeq® 500/550 mid output kit v2 chemistry (300 cycles) (Illumina, Eindhoven, Netherlands).

Sequencing reads of *Bordetella* strains 3136, 3227, 3561 and 3562 were quality clipped with Trimmomatic^34^ and afterwards *de novo* assembled with SPAdes^35^. Resulting scaffolds were oriented onto the following *Bordetella* genomes: *B. pseudohinzii* strain 8–296–03 (JHEP02000048*)*, *B. hinzii* LMG 13501 (LRUJ00000000), *B. avium* 197N^T^ (NC_010645), *B. holmesii* ATCC 51541^T^ (NZ_CP007494*)*, *B. pertussis* Tohama IT (BX470248), *B. petrii* DSM 12804 (NC_010170) and *B. trematum* H044680328^T^ (NZ_LT546645) with Contiguator^36^. Subsequently, pseudo-genomes were annotated via Prokka^36^.

Sequencing reads were quality clipped as described before and mapped onto aforementioned *Bordetella* genomes with bowtie2^37^. Afterwards, SNPs were detected via Samtools and BCFtools toolkits^38^. Called SNPs were filtered with SnpSift^38,39^ and finally annotated with SnpEff^40^. Finally, for comparative analyses GECO^17^ and EDGAR^16^ were used with the following genomes: *B. avium* 197N^T^ (NC_010645), *B. hinzii* F582^T^ (NZ_CP012076), *B. hinzii* LMG 13501 (LRUJ00000000), *B. holmesii* ATCC 51541^T^ (NZ_CP007494), *B. holmesii* H558 (NZ_CP007158), *B. pertussis* Tohama I^T^ (BX470248), *B. petrii* DSM 12804^T^ (NC_010170), *B. pseudohinzii* 8–296–03 (JHEP02000048), *B. pseudohinzii* HI4681^T^ (NZ_CP016440), *B. trematum* CCUG 13902 (AWNL01000001), *B. trematum* CCUG H044680328^T^ (NZ_LT546645) as well as aforementioned *Bordetella* strains 3136, 3227, 3561 *and* 3562.

### DNA isolation and PCR

Genomic DNA was isolated from faecal and larynx samples (infected and non-infected, each n = 4) and cultured *B. pseudohinzii* (n = 1) with the ISOLATE Faecal DNA Kit (Bioline, Luckenwalde, Germany) according to the manufacturer’s protocol. For subsequent PCR, 3 µl of DNA, 2.5 µl buffer II (100 mM Tris-HCl, 500 mM KCl, pH 8.3), 2 µl MgCl_2_ (15 mM), 0.75 µl dNTP (10 mM each), 0.75 µl of each primer (10 µM; fwd: cgttccggttgcccagaag, rev: gccttcggcggtcttgttggtc; accession number CP016440; Eurofins, Ebersberg, Germany), 0.25 µl AmpliTaq Gold polymerase (5 U/µl; if not otherwise indicated, all reagents from Perkin Elmer, Langen, Germany) were supplemented with H_2_O to a final volume of 25 µl. Cycling conditions were 12 min 95 °C, 40 cycles with 30 s at 95 °C, 30 s at 62 °C, 30 s at 72 °C, and a final extension at 72 °C for 7 min. PCR products were analysed on a 2% agarose gel. For subsequent sequencing, PCR-products were purified by the use of the Monarch DNA Gel Extraction Kit (New England Biolabs, Frankfurt, Germany) as recommended by the manufacturer. Sequencing was done by Eurofins.

### Histopathology

Mice were sacrificed by cervical dislocation and the lungs were removed after ligation of the trachea to avoid alveolar collapse as described^41^. Lungs were immersion fixed for at least 12 h in 4% paraformaldehyde (Carl Roth) in 0.1 M phosphate buffer, washed in 0.1 M phosphate buffer and embedded in paraffin, cut in 2 µm thick horizontal sections and stained with hematoxylin and eosin (H&E) after dewaxing in xylene and rehydration in decreasing ethanol concentrations. An additional serial section was Giemsa-stained for visualization of bacterial DNA. Two pathologists (CG, KD) evaluated three evenly distributed sections per lung microscopically to assess the distribution and quality of pathological alterations using specified inflammation parameters such as tracheitis, total lung area affected, distribution of lung lesions, peribronchial, interstitial and intra-alveolar inflammation, infiltration by neutrophils, macrophages and the presence of BALT and lymphoid hyperplasia of lymph nodes.

### Counting of ciliated tracheal epithelial cells

To quantify the number of ciliated cells, the tracheas of C57BL/6 J mice were removed and fixed by immersion for at least 12 h in Zamboni fixative (2% formaldehyde [Roth] and 15% picric acid [Merck] in 0.1 M phosphate buffer). Afterwards, specimens were washed, paraffin embedded and 7 µm thick coronal sections were cut and stained with H&E. At least 400 nucleated epithelial cell profiles per animal were classified as belonging to either ciliated or non-ciliated cells; images were taken using a LEICA DM750 light microscope (Bensheim, Germany).

### Cell counting in BAL fluid

Animals were sacrificed, the trachea was exposed and cannulated. Lungs were flushed twice with 800 µl of sterile PBS (Thermo Fisher Scientific, Waltham, USA) each. Cytospins (Cellspin® I, Cellspin diagnostics, Waldsolms, Germany) were prepared from BAL fluid and Pappenheim-stained (Merck, Darmstadt, Germany). Differential cell counts were performed from at least 250 cells per sample using a Neubauer chamber.

### Cultivation of B. pseudohinzii isolates

Isolates of *B. pseudohinzii* were cultured overnight at 37 °C in BHI medium (Thermo Fisher Scientific). Thereafter, bacteria were diluted and cultured until they reached a phase of logarithmic growth, followed by three times washing with minimal essential medium (MEM; Thermo Fisher Scientific) without antibiotics. Bacterial infection dose was determined by plating bacterial suspension on BHI agar plates and counting CFU after 12 h of incubation at 37 °C.

### *In vitro*-infection of tracheal explants

Tracheas of *B. pseudohinzii-*negative C57BL/6 J mice were explanted aseptically, surrounding tissue was removed and tracheas were cut into approximately 2 mm long pieces consisting of 2–3 cartilage rings. For electron microscopy, the rings were opened by cutting the trachealis muscle, washed in sterile PBS (Thermo Fisher Scientific) supplemented with penicillin (100 U/ml; PAA, Etobicoke, Canada) and streptomycin (0.1 mg/ml; PAA) and transferred into 6-well culture plates (Greiner bio–one, Kremsmünster, Austria). The rings were then submerged in phenol red–free MEM (Thermo Fisher Scientific) supplemented with penicillin, streptomycin and L-glutamine (2 mM) (Thermo Fischer Scientific) and cultured at 37 °C and 5% CO_2_ for 24 h. After 24 h, viability of explants was validated by visualizing the beating of ciliated cells. Vital explants were then transferred into fresh plates and washed with subsequent addition of *B. pseudohinzii* cultures (strain 3227). Throughout, explants were kept in 1 ml of medium.

For measurements of PTS and CBF of *in vitro-*infected tracheal explants, each trachea was divided into 2 halves. They were transferred into a delta T-dish, washed with PBS, bacteria were added and the samples were incubated for 4 h at 37 °C. PTS and CBF were examined as described below. Throughout, explants were kept in 1 ml of medium.

### Electron microscopy

Tracheal explants were fixed for at least 4 h in a fixative mixture consisting of 1.5% glutardialdehyde (Merck) and 2.5% paraformaldehyde in 0.1 M phosphate buffer (pH 7.4). After fixation, tissues were washed in HEPES, osmicated for 2 h in aqueous 1% osmium tetroxide (Sigma Aldrich) and washed in distilled water. For scanning electron microscopy, samples were dehydrated, critical point dried and sputtered with gold. Samples were examined by using Phillips XL30 and Zeiss EM9DSM982 scanning electron microscopes. For transmission electron microscopy, specimens were contrasted in 1% uranyl acetate (Merck) overnight, dehydrated and embedded in epon (Sigma Aldrich, St. Louis, USA). Sections of 80 nm thickness were cut using an ultramicrotome (Reichert Ultracut E, Leica, Bensheim, Germany), stained with alkaline lead citrate and viewed using a EM 902 (Zeiss, Wetzlar, Germany) transmission electron microscope equipped with a slow scan 2 K CCD camera (TRS; Tröndle, Moorenweis, Germany). Semithin sections of 0.8 µm thickness were cut using the same ultramicrotome and stained with methylene blue (Carl Roth, Karlsruhe, Germany) for light microscopic evaluation (LEICA DM750, Bensheim, Germany).

### Negative staining of B. pseudohinzii

*B. pseudohinzii* (strain 3227) was cultured in BHI medium, fixed with 4% paraformaldehyde solution and washed in 0.1 M phosphate buffer. This bacterial suspension was placed on a Formvar-coated grid (Plano GmbH, Wetzlar, Germany), stained with 1% ammonium heptamolybdate (Merck) for 30 s, and analysed by transmission electron microscopy (EM 902).

### Measurement of PTS

A modified version of the previously published method was used^15^. Briefly, the explanted trachea was transferred in a delta T-dish with a thin layer of Sylgard polymer (Dow Corning, Wiesbaden, Germany) on its glass bottom. After the surrounding tissue was removed, tracheas were oriented with the *m. trachealis* facing upward, fixed with insect needles and submerged in HEPES (pH 7.4). The temperature of the buffer was constantly held at 31 °C. The muscle was cut and the trachea was fixed flat on the T-dish. Polysterene dynabeads with a diameter of 2.8 µm (Invitrogen, Carlsbad, USA) were added before measurements.

For the freshly explanted tracheas, the first measurement was taken 30 min after the mouse was sacrificed, followed by measurements every 5 min until 55 min after start, then every 2 min. ATP (100 µM) was added at minute 59. Drugs and dynabeads were well mixed before every measurement. For measuring the baseline PTS of *in vitro-*infected tracheas, three videos of three different areas were taken after 2 min of equilibration time. For each measurement, videos consisting of 200 images (640 × 512 pixels; 12 bit; 1 image per 84 ms) were taken with using UMPLFL20xW/0.5 water immersion objective (Olympus, Shinjuku, Japan) and a SMX-150M (EHD imaging GmbH, Damme, Germany) camera. Dynabeads were tracked and visualized by using Image-Pro Plus (MediaCybernetics, Rockville, USA) software, which calculated the average speed of all tracked particles.

### Measurement of CBF

Preparation of tracheas and microscope setup were the same as for PTS measurements, except that images were taken at higher magnification (UMPLFL40xW/0.8 water immersion objective; Olympus, Shinjuku, Japan). Videos were taken before adding the dynabeads for the PTS measurements. For each animal, one video was taken. To investigate the baseline CBF of *in vitro*-infected tracheas, two videos of two different areas were taken after 2 min of equilibration time. The videos consisted of 1000 images with a frame rate of 105 images/s. During the measurements, no drugs were added, in order to determine a baseline CBF. For calculation of mean CBF, the dominating beat frequency of each single ciliated cell (30–45 cells per video) was analysed by fast Fourier transformation using a Graphical User Interface (GUI) for MATLAB R2016b which was programmed by Peter König (University of Lübeck, Germany).

### Statistical Analysis

Data are presented as individual data points in figures and as mean ± standard error of the mean (SEM). Statistical analyses of data were performed with GraphPad Prism version 6 (La Jolla, USA). Data were analysed for normal distribution by the Kolmogorov-Smirnov test followed by two tailed Student’s unpaired t-test.

### Data Availability

Sequence data for *B. pseudohinzii* (3136, 3227, 3561 and 3562) have been submitted to the NCBI Short Read Archive repository under the SRA accession numbers SRP133109 (https://www.ncbi.nlm.nih.gov/sra/SRP133109), SRP133061 (https://www.ncbi.nlm.nih.gov/sra/SRP133061), SRP133197 (https://www.ncbi.nlm.nih.gov/sra/SRP133197) and SRP133199 (https://www.ncbi.nlm.nih.gov/sra/SRP133199).

## Electronic supplementary material

Supplementary Information
