# Supplementary material for: Bordetella pseudohinzii targets cilia and impairs tracheal cilia-driven transport in naturally acquired infection in mice
Source: Sci Rep. 2018 Apr 9;8:5681. doi: 10.1038/s41598-018-23830-4 (PMC5890243; doi:10.1038/s41598-018-23830-4)
Supplement: Supplementary file 1 — Supplementary Information [file 41598_2018_23830_MOESM1_ESM.docx]

*Bordetella pseudohinzii* targets cilia and impairs tracheal cilia-driven transport in naturally acquired infection in mice

Alexander Perniss^1^*, Nadine Schmidt^2^, Corinne Gurtner^3^, Kristina Dietert^3^, Oliver Schwengers^4,5,6^, Markus Weigel^4^, Julia Hempe^7^, Christa Ewers^2^, Uwe Pfeil^1^, Ulrich Gärtner^1^, Achim D. Gruber^3^, Torsten Hain^4,6#^& Wolfgang Kummer^1#^

^1^Institute of Anatomy and Cell Biology, German Center for Lung Research (DZL), Excellence Cluster Cardio-Pulmonary System (ECCPS), Justus-Liebig-University Giessen, Germany; ^2^Institute of Hygiene and Infectious Diseases of Animals, Justus-Liebig-University Giessen; ^3^Institute of Veterinary Pathology, Freie Universität Berlin, Berlin, Germany; ^4^Institute for Medical Microbiology, Justus-Liebig-University Giessen; ^5^Bioinformatics and System Biology, Justus Liebig University, Giessen; ^6^German Center for Infection Research (DZIF), Partner Site Giessen-Marburg-Langen, Giessen; ^7^Central Experimental Animal Facility, Justus-Liebig-University Giessen

^#^Authors contributed equally

*Corresponding author: Alexander Perniss; email: Alexander.Perniss@anatomie.med.uni-giessen.de; phone number: +49 6419947004; fax number: +49 6419947009; address: Institute of Anatomy and Cell Biology, Justus-Liebig-University Giessen, Aulweg 123, 35385 Giessen


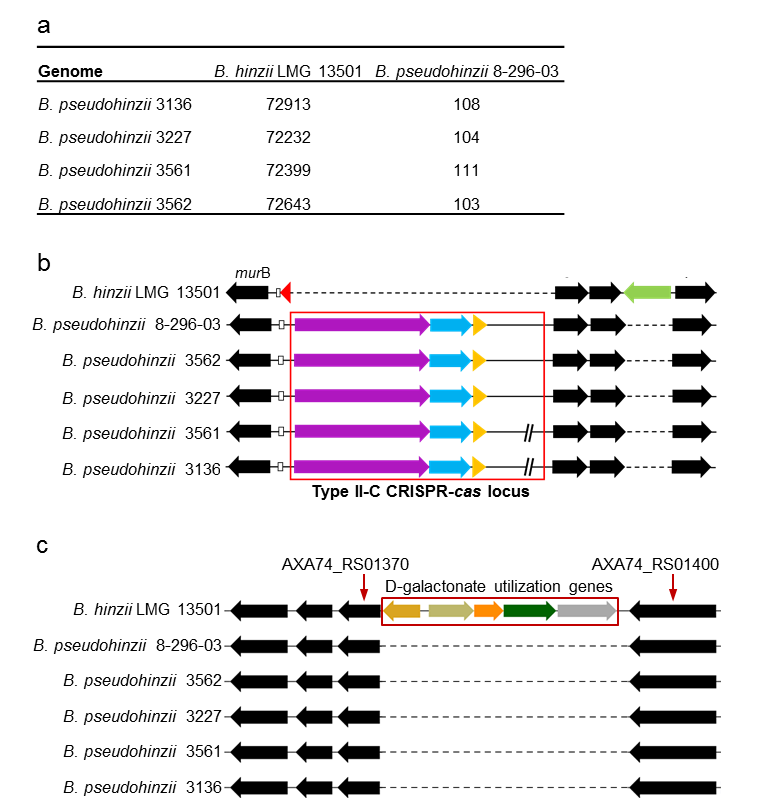


Supplementary Figure 1

Differences in the genomes of *B. hinzii* and *B. pseudohinzii*. (**a**) Number of single nucleotide polymorphisms in the four sequenced genomes of *B. hinzii* and *B. pseudohinzii.* (**b**) Chromosomal overview of the Type II-C CRISPR-*cas* locus (red box) in *B. pseudohinzii* compared to *B. hinzii*. The dotted line indicates the absence of the Type II-C CRISPR-*cas* locus in *B. hinzii*. Core genes (homologous genes [>87% identity / >99% coverage] among all compared genomes) are depicted in black. Contig borders are indicated as breaks for *B. pseudohinzii* 3561 and 3161 (**c**) Chromosomal overview of the D-galactonate utilization gene region (red box) in *B. hinzii* compared to *B. pseudohinzii*. The dotted line indicates the absence of the D-galactonate utilization locus in *B. pseudohinzii*. Core genes (homologous genes [>95% identity / >90% coverage] among all compared genomes) are depicted in black.

**
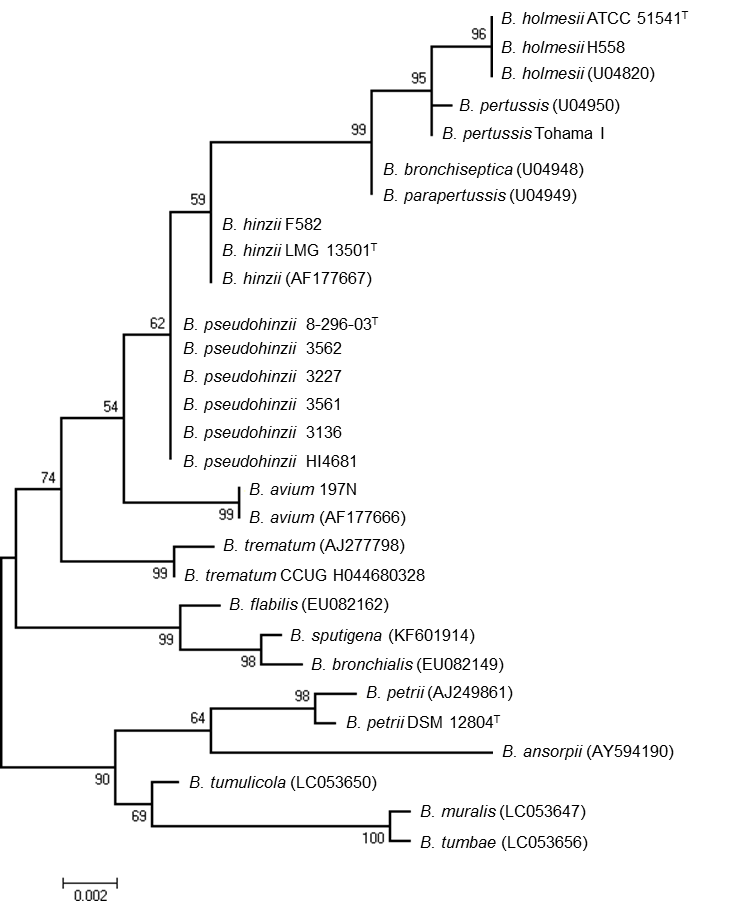
**

**Supplementary Figure 2**

Phylogenetic tree based on 16S rRNA gene sequences of *Bordetella* species. The tree was constructed using the neighbour-joining method. Bootstrap values of 1000 repeats are shown next to the branches. 16S rRNA sequence accession numbers are indicated in brackets, otherwise taken from RefSeq database (https://www.ncbi.nlm.nih.gov/refseq/).


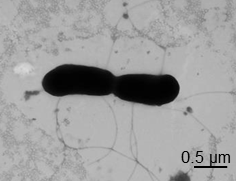


**Supplementary Figure 3**

Transmission electron microscopic image of negative stained *B. pseudohinzii* (strain 3227). Bacteria are displayed as rod-shaped coccobacilli with several flagella.


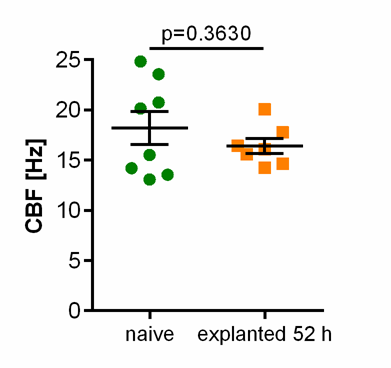


Supplementary Figure 4

Cultivation of tracheal explants does not change ciliary beat frequency (CBF). CBF was measured 27 min after explantation (green circles; n=7) or following a culture period of 52 h (orange squares; n=7). Data are shown as individual points and mean±SEM. No significant difference in CBF was observed, Student´s unpaired t-test.


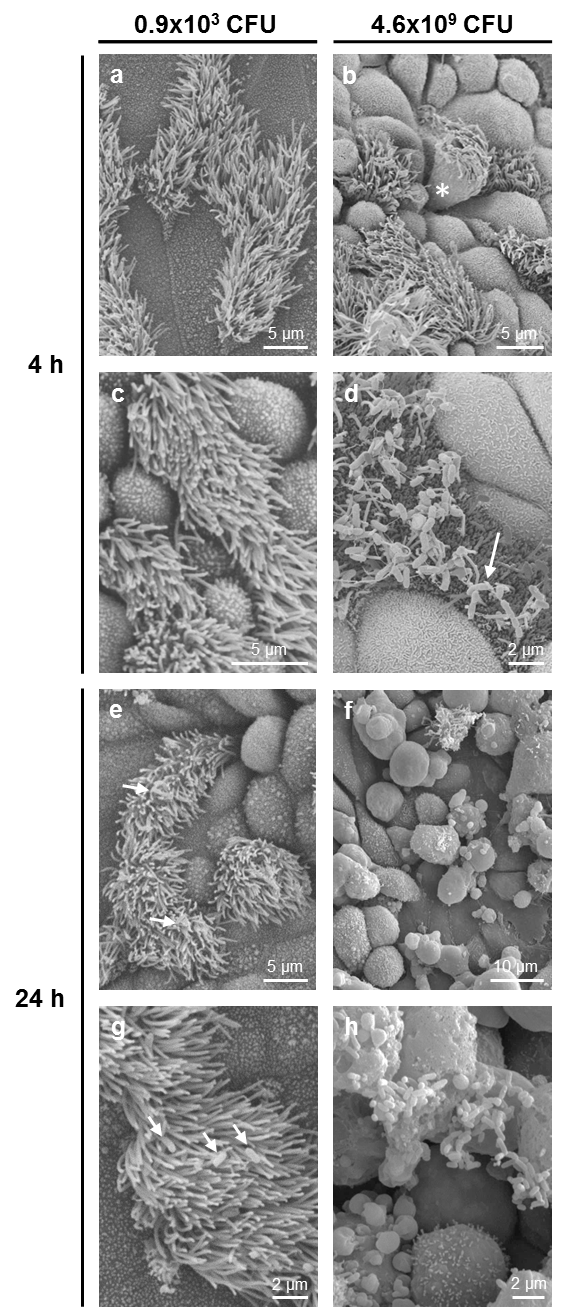


Supplementary Figure 5

**Supplementary Figure 5.** *B. pseudohinzii* (strain 3227) attaches to cilia and damages the epithelium in an *in vitro*-infection model. Tracheal explants were cultured for 24 h, with following addition of bacteria for 4 h or 24 h, and analysed by scanning electron microscopy. (**a,c**) Cultivation for 4 h with the lowest dose of *B. pseudohinzii* used in the experiments (0.9x10^3^ CFU). Cilia are regularly orientated and no bacteria are visible. (**b,d**) Cultivation for 4 h with the highest dose of *B.* *pseudohinzii* used in the experiments (4.6x10^9^ CFU). (**b**) Single ciliated cells detach from the epithelium (asterisk) as an indicator of epithelial damage. (**d**) Cilia of individual ciliated cells are shortened and reduced in number, single bacteria attach to every cilium (*arrow*). (**e,g**) Cultivation for 24 h with 0.9x10^3^ CFU. Single bacteria attach to cilia (arrows), cilia are regularly orientated and no damage of the epithelium can be observed. (**f,h**) Cultivation for 24 h with 4.6x10^9^ CFU *B. pseudohinzii*. Only remnants of ciliated cells are present and blebbing of cells as an indicator of apoptosis takes place. Bacteria form microcolonies.

**
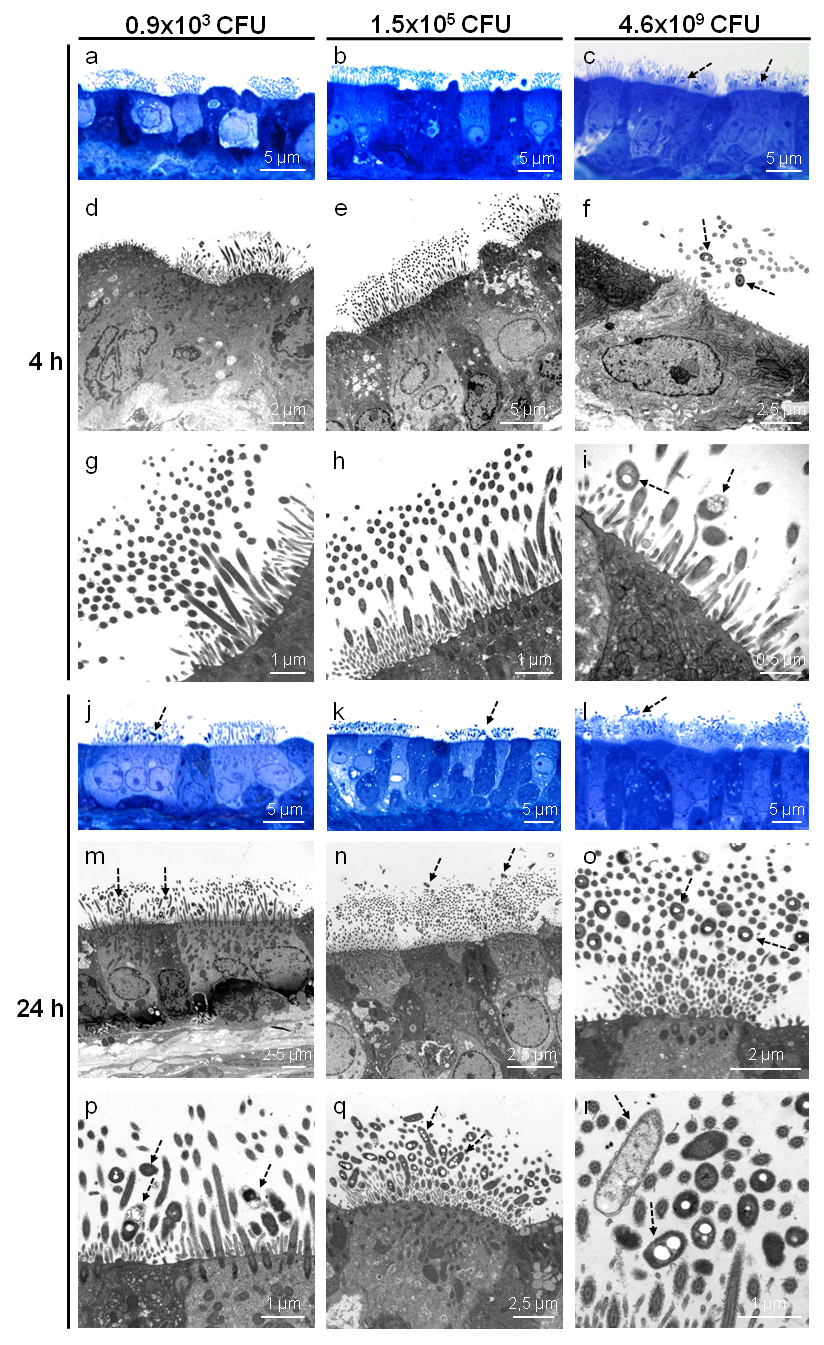
**

Supplementary Figure 6

**Supplementary Figure 6.** *B. pseudohinzii* (strain 3227) attaches to and intermingles with cilia in an *in vitro*-infection model. Tracheal explants were cultured for 24 h, with following addition of bacteria for 4 h (**a-i**) or 24 h (**j-r**). (**a-c**) Semithin sections of tracheal explants cultured for 24+4 h, light microscopy. (**a**) *B. pseudohinzii* (0.9x10^3^ CFU) was added for 4 h. No bacteria can be found, cilia are normally orientated. (**b**) *B. pseudohinzii* (1.5x10^5^ CFU) was added for 4 h. No bacteria are seen. (**c**) *B. pseudohinzii* (4.6x10^9^ CFU) was added for 4 h. Bacteria attach to cilia in high number (*dotted arrows*). (**d-i**) Transmission electron microscopy, tracheas were cultured for 4 h with bacteria. (**d,g**) *B. pseudohinzii* (0.9x10^3^ CFU) was added, no bacteria were found. (**e,h**) *B. pseudohinzii* (1.5x10^5^ CFU) was added, no bacteria were found, cilia are inconspicuous. (**f,i**) *B. pseudohinzii* (4.6x10^9^ CFU) was added, bacteria intermingle with cilia (*dotted arrows*). (**j-l**) Semithin sections of tracheal explants cultured for 24+24 h, light microscopy. (**j**) Bacteria were added for 24 h (0.9x10^3^ CFU). Bacteria are located between cilia (*dotted arrows*), no bacteria can be observed on non-ciliated cells. (**k**) Bacteria were added for 24 h (1.5x10^5^ CFU). High numbers of bacteria attach to cilia (*dotted arrows*). (**l**) Bacteria were added for 24 h (4.6x10^9^ CFU). Bacteria attach to cilia. Microcolonies of bacteria are located on top of the cilia (*dotted arrows*). (**m-r**) Transmission electron microscopy, tracheas were cultured for 24 h with bacteria. (**m,p**) *B. pseudohinzii* (0.9x10^3^ CFU) was added. Bacteria attach to cilia (*dotted arrows*), no damage of the epithelium can be observed. (**n,q**) Bacteria were added (1.5x10^5^ CFU). Bacteria intermingle with cilia, dividing bacteria can be observed (*dotted arrow*; **q**). (**o,p**) *B. pseudohinzii* (4.6x10^9^ CFU) was added. Numerous bacteria are located between the cilia (*dotted arrows*).


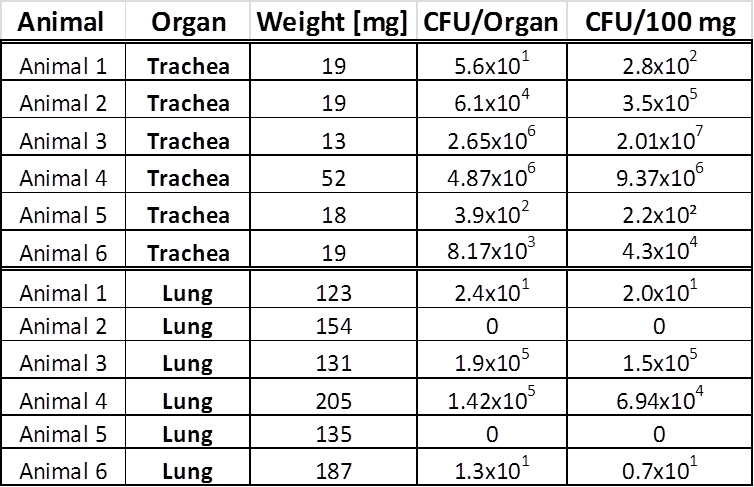


**Supplementary Table 1**

Colony forming units (CFU) of *Bordetella pseudohinzii* grown from tissue homogenates of whole trachea and lung from 6 animals. Values are presented as CFU per organ and as CFU per 100 mg tissue.

|  |  | Raw | | | QC | | |
| --- | --- | --- | --- | --- | --- | --- | --- |
| Genome | Direction | # Reads | Length | Quality | **# Reads** | **Length** | **Quality** |
|  |  |  | min  mean  max | min  mean  max |  | min  mean  max | min  mean  max |
| *B. pseudohinzii 3136* | fw | 4,642,104 | 35  123.6  151 | 30  34  35 | 4,048,243 | 20  119.7  151 | 31  34.7  35 |
|  | rv |  | 35  125.7  151 | 26  32  33 |  | 20  110  151 | 31  33.8  34 |
| *B. pseudohinzii 3227* | fw | 2,150,115 | 35  121.1  151 | 30  34  35 | 1,876,635 | 20  117.7  151 | 31  34.6  35 |
|  | rv |  | 35  123.4  151 | 26  32  33 |  | 20  108.5  151 | 31  33.8  34 |
| *B. pseudohinzii 3561* | fw | 1,833,587 | 35  130.2  151 | 27  32.9  35 | 1,525,979 | 20  119.3  151 | 31  33.9  35 |
|  | rv |  | 35  131.7  151 | 24  30.8  33 |  | 20  105.6  151 | 30  32.9  34 |
| *B. pseudohinzii 3562* | fw | 2,159,942 | 35  123.1  151 | 30  34.1  35 | 1,908,654 | 20  119.8  151 | 31  34.7  35 |
|  | rv |  | 35  124.9  151 | 26  32.2  33 |  | 20  110.8  151 | 31  33.9  34 |

**Supplementary Table 2**

Sequencing and quality metrics of sequenced isolates. Note: fw/rv denote direction of paired-end sequencing reads, i.e. forward direction and reverse direction, respectively; raw: metrics given for raw sequencing reads; QC: metric results after adapter trimming and quality clipping; quality values are provided as Phred scores.

| Genome | # contigs | N50 coverage | N50 length | Genome size | GC |
| --- | --- | --- | --- | --- | --- |
| *B. pseudohinzii 3136* | 60 | 78 | 274,686 | 4,538,910 | 67 |
| *B. pseudohinzii 3227* | 71 | 34 | 144,730 | 4,537,073 | 67 |
| *B. pseudohinzii 3561* | 77 | 29 | 144,702 | 4,537,656 | 67 |
| *B. pseudohinzii 3562* | 76 | 36 | 151,848 | 4,538,016 | 67 |

**Supplementary Table 3.**

Assembly statistics of sequenced isolates. Note: N50 coverage: length weighted arithmetic mean coverage of all contigs which lengths are equal or larger than the N50 length. Thereby, this coverage metric is more robust against outliers mostly posing short to very short contigs with very high coverage; GC: values given in percent.
